# Oxidative Stress Parameters Can Predict the Response to Erythropoiesis-Stimulating Agents in Myelodysplastic Syndrome Patients

**DOI:** 10.3389/fcell.2021.701328

**Published:** 2021-06-07

**Authors:** Ana Cristina Gonçalves, Raquel Alves, Inês Baldeiras, Joana Jorge, Bárbara Marques, Artur Paiva, Bárbara Oliveiros, Emília Cortesão, José Manuel Nascimento Costa, Ana Bela Sarmento-Ribeiro

**Affiliations:** ^1^University of Coimbra, Laboratory of Oncobiology and Hematology and University Clinic of Hematology, Faculty of Medicine (FMUC), Coimbra, Portugal; ^2^University of Coimbra, Group of Environment, Genetics and Oncobiology (CIMAGO) – Institute for Clinical and Biomedical Research (iCBR), FMUC, Coimbra, Portugal; ^3^University of Coimbra, Center for Innovative Biomedicine and Biotechnology (CIBB), Coimbra, Portugal; ^4^Clinical Academic Center of Coimbra (CACC), Coimbra, Portugal; ^5^University of Coimbra, FMUC, Coimbra, Portugal; ^6^University of Coimbra, Center for Neuroscience and Cell Biology, Coimbra, Portugal; ^7^Clinical Hematology Department, Centro Hospitalar e Universitário de Coimbra (CHUC), Coimbra, Portugal; ^8^Centro Hospitalar e Universitário de Coimbra (CHUC), Unidade de Gestão Operacional em Citometria, Coimbra, Portugal; ^9^University of Coimbra, University Clinic of Oncology, FMUC, Coimbra, Portugal

**Keywords:** myelodysplastic syndrome, erythropoiesis-stimulating agents, response biomarker, oxidative stress, antioxidant defenses, reactive oxygen species

## Abstract

Oxidative stress has been implicated in the development of several types of cancer, including myelodysplastic syndromes (MDS), as well as in the resistance to treatment. In this work, we assessed the potential of oxidative stress parameters to predict the response to erythropoiesis-stimulating agents (ESAs) in lower-risk MDS patients. To this end, we analyzed the systemic levels of reactive species (peroxides and NO), antioxidant defenses (uric acid, vitamin E, vitamin A, GSH, GSSG, TAS, as well as GPX and GR activities], and oxidative damage (8-OH-dG and MDA) in 66 MDS patients, from those 44 have been treated with ESA. We also calculated the peroxides/TAS and NO/TAS ratios and analyzed the gene expression of levels of the redox regulators, NFE2L2 and KEAP1. We found that patients that respond to ESA treatment showed lower levels of plasma peroxides (*p* < 0.001), cellular GSH (*p* < 0.001), and cellular GR activity (*p* = 0.001) when compared to patients who did not respond to ESA treatment. ESA responders also showed lower levels of peroxides/TAS ratio (*p* < 0.001) and higher levels of the expression of the NFE2L2 gene (*p* = 0.001) than those that did not respond to ESA treatment. The levels of plasmatic peroxides shown to be the most accurate biomarker of ESA response, with good sensitivity (80%) and specificity (100%) and is an independent biomarker associated with therapy response. Overall, the present study demonstrated a correlation between oxidative stress levels and the response to ESA treatment in lower-risk MDS patients, with the plasmatic peroxides levels a good predictive biomarker of drug (ESA) response.

## Introduction

Myelodysplastic syndromes (MDS) are clonal hematological malignancies that comprises several subtypes with different biological and clinical presentations. These diseases are frequently characterized by inefficient hematopoiesis, dysplasia in one or more myeloid cell lineages, variable degrees and number of cytopenias, and increased risk of progression to acute myeloid leukemia (AML) (Jhanwar, 2015; Cazzola, 2020). MDS blood show typical morphological features such as dysplasia, differentiation arrest, defective cellular functions, and genomic instability. The peripheral cytopenias frequently observed in these patients are a consequence of the ineffective hematopoiesis and could involve all myeloid lineages (Mufti, 2004). One of the MDS paradoxes is the coexistence of peripheral cytopenias with hyperplastic bone marrow. The MDS clones exhibit increased proliferation, however, this proliferation is rapidly balanced by increased apoptosis, namely in low risk MDS subtypes. Recently, precursors conditions of MDS, such as clonal hematopoiesis of indeterminate potential (CHIP) and clonal cytopenia of undetermined significance (CCUS), has been identified (Cazzola, 2020) that allow an earlier diagnosis and could modify the MDS approach.

The presence of cytopenia and dysplasia, in at least one hematologic cell line, are essential for MDS diagnosis (Cazzola, 2020). On the other hand, the risk of death from cytopenias complications or evolution to AML is highly variable in MDS patients. The Revised International Prognostic Scoring System (IPSS-R) is the most frequently used prognostic system and is based on a small number of features with independent prognostic value, including chromosomal abnormalities, bone marrow blasts, hemoglobin level, platelet count and absolute neutrophil count. These features are routinely available in all clinical centers and allow the classification of MDS patients in five risk subgroups (very low risk, low risk; intermediate, high risk, and very high risk) with different probabilities of AML progression and survival (Garcia-Manero, 2010; Adès et al., 2014; Cazzola, 2020). In clinical practice, clinicians usually distinguish patients in lower-risk MDS (IPSS-R risk score ≤ 3.5) and in higher-risk MDS (IPSS-R risk score > 3.5), and lower-risk MDS account for about two-thirds of all MDS cases (Komrokji et al., 2011; Cazzola, 2020). In lower-risk MDS patients with symptomatic anemia the first-line treatment option is erythropoiesis-stimulating agents (ESAs), namely recombinant human erythropoietins (rHuEPOs), alone or combined with granulocyte colony-stimulating factor (G-CSF), which could prevent or delay transfusion dependency and improve quality of life. However, these treatments are only recommended for patients with serum erythropoietin (sEPO) levels below 500 U/L, and the most reliable predictor of a response is a sEPO lower than 200 U/L, while a high transfusion requirement predicts treatment failure (Cazzola, 2020; Park et al., 2020). However, in clinical practice, a sEPO cutoff level of 200 U/L is more indicative of response (Park et al., 2020). New biomarkers and scoring systems should be investigated to improve response and/or to predict resistance to ESA therapy.

Reactive oxygen species (ROS) are relevant players in hematological malignancies development, progression, and therapy resistance (Sardina et al., 2012; Sarmento-Ribeiro et al., 2012; Gonçalves et al., 2015). These free radical molecules show both beneficial and deleterious consequences (Ghaffari, 2008; Imbesi et al., 2013). When ROS levels overcome the cellular antioxidant defenses, oxidative stress is established as result of an imbalance in redox homeostasis (Ghaffari, 2008; Sardina et al., 2012). Several signaling pathways such as proliferation, differentiation, and apoptosis are regulated by intracellular ROS levels (Imbesi et al., 2013; Hasselbalch et al., 2014). Blood cells from MDS patients show increased levels of ROS and decreased concentration of GSH (Ghoti et al., 2007; Gonçalves et al., 2015). Furthermore, the disruption of redox homeostasis is a crucial factor in drug resistance development, which is an important factor in the failure of anticancer (Liu et al., 2016). During erythroid differentiation, erythroblasts are exposed to an oxidative environment and ROS are produced in response to EPO (Zhao et al., 2016; Beneduce et al., 2019). These ROS could act as second messengers by modulating intracellular signaling kinases including JAK2, LYN, and FYN (Beneduce et al., 2019). Beneduce et al. (2019) found that in the absence of FYN kinase, the efficiency of the EPO signal is decreased and an increase in ROS production is observed (Beneduce et al., 2019). However, the dynamics of oxidative status during erythropoiesis and erythroid differentiation in response to EPO are still unclear. Additionally, the nuclear factor erythroid 2-related factor 2 (NRF2), a major cellular redox modulator, plays a crucial role in preventing cancer cells from cytotoxicity induced by chemotherapy, contributing to drug resistance and therapeutic failure. In higher-risk MDS patients, NRF2 expression showed a significant prognostic value for overall survival being associated with cytarabine resistance (Lin et al., 2019). In this context, we investigate the potential of oxidative stress parameters as predictive biomarkers of response to ESA treatment in MDS patients.

## Materials and Methods

### Study Population

This study enrolled 66 MDS patients at diagnosis from October 2012 to March 2014. MDS patients were diagnosed according to the World Health Organization 2016 classification of myeloid neoplasms (Arber et al., 2016) in the following subtypes: MDS with single lineage dysplasia (MDS-SLD), MDS with multilineage dysplasia (MDS-MD), MDS with ring sideroblasts (MDS-RS), and MDS with excess blasts (MDS-EB). Patients were stratified according to IPSS-R in lower risk and higher-risk patients (Cazzola, 2020). Biodemographic (age and gender) and clinical data, when available, were obtained from medical records.

The Ethics Committee of the Faculty of Medicine of the University of Coimbra (Coimbra, Portugal) approved the research procedures, and the study was conducted following the Declaration of Helsinki. Before enrollment, participants provided their informed consent for participation. The international ethical guidelines of confidentiality, anonymity of personal data, and abandonment option, in case of expressed, will were be followed.

### Sample Preparation

Peripheral blood samples were collected at diagnosis, after fasting, into sodium heparin tubes. Samples were immediately centrifuged; plasma and red blood cells (with a concentration of hemoglobin adjusted at 100 g/l) were stored frozen at -20°C until analysis, as previously described (Baldeiras et al., 2010). Some oxidative stress parameters were normalized to total plasma cholesterol.

### Uric Acid Determinations

Plasmatic levels of uric acid were determined by a colorimetric method (Barham and Trinder, 1972) based on the reduction of uric acid by the uricase enzyme. In this reaction, hydrogen peroxide is released and forms a chromogenic compound evaluated spectrophotometrically at 550 nm.

### Vitamin A and E Measurements

The plasmatic levels of vitamins A (vit A) and E (vit E) were assessed in lipid extracts obtained from plasma samples and quantified by high-performance liquid chromatography (HPLC) using an analytic column spherisorb ODS1-5 μm (250 mm × 4.6 mm), eluted at 2.5 ml/min with a water solution of methanol (90%), at 45°C, and detected in a spectrophotometer (Gilson) at 340 nm (for vit A) or 295 nm (for vit E). The erythrocytic vitamin E content was extracted in *n*-hexane and quantified by reverse-phase HPLC (Vatassery et al., 1978; De Leenheer et al., 1979), using an analytic column spherisorb S10w (250 mm × 4.6 mm), eluted at 1.5 ml/min with *n*-hexane modified with 0.9% of methanol, and detected by spectrophotometry at 287 nm (Gilson).

### Oxidized and Reduced Glutathione Quantification

The erythrocytic reduced glutathione (GSH) and oxidized glutathione (GSSG) were also evaluated by HPLC with fluorimetric detection (excitation at 385 nm, and emission at 515 nm), using the Immunodiagnostik kit (Immunodiagnostik AG, Bensheim, Germany), as described by the manufacturer.

### Antioxidant Enzymes Activity Determination

The glutathione peroxidase (GPX) activity in red blood cells was evaluated by spectrophotometry using an indirect determination method and *tert*-butyl hydroperoxide as substrate (Paglia and Valentine, 1967). The GSSG formation was examined through the quantification of reduced nicotinamide adenine dinucleotide phosphate (NADPH) oxidation at 340 nm in a thermostated spectrophotometer UVIKON 933 UV/Visible. The activity of glutathione reductase (GR) in red blood cells was evaluated by spectrophotometry at 340 nm (Goldberg and Spooner, 1983), using GSSG as a substrate, and monitoring its reduction to GSH through the assessment of NADPH oxidation at 37°C in a spectrophotometer UVIKON 933 UV/Visible.

### Plasmatic Peroxide Quantification

The plasmatic levels of peroxides were quantified using the kit Thermo Scientific Pierce Quantitative Peroxide Assay Kit – lipid-compatible formulation (Life Technologies), according to the manufacturer, in a Synergy^TM^ multi-mode microplate reader (BioTek Instruments).

### Total Antioxidant Status Evaluation

The plasmatic total antioxidant status (TAS) was assessed by a chromogenic method (Randox Laboratories) based on the plasma capacity to inhibit the formation of the ABTS^+^ radical cation (2,2′-azino-di-[3-etilbenzotiazolin sulfonate]), and detected at 600 nm as described by the manufacturer.

### Lipid Peroxidation Measurements

The plasmatic and erythrocytic lipid peroxidation were assessed by the formation of thiobarbituric acid (TBA) adducts of malondialdehyde (MDA), separated by HPLC (Gilson), and quantified fluorimetrically using the ClinRep complete kit (RECIPE), as described by the manufacturer. Briefly, 100 μl blank, standard, controls, and patients’ samples were first derivatized at 100°C for 60 min in a glass light-protected vial. After cooling, samples were neutralized, precipitated, and centrifuged at 10,000 *g* for 5 min. Finally, 20 μl of the supernatants were injected into the HPLC and the MDA adducts were determined fluorimetrically (excitation at 515 nm, and emission at 553 nm; FP-2020/2025, Jasco, Tokyo, Japan).

### Plasmatic Nitric Oxide Quantification

The plasmatic levels of nitric oxide (NO) were determined by a photometric method (Roche Diagnostics GmbH) based on the detection of its oxidation products, nitrite and nitrate (Titheradge, 1998). First, the nitrate present in the ultra-filtrated plasma was reduced to nitrite, which then reacted with sulphanilamide and *N*-(1-naphthyl)-ethylenediamine dihydrochloride to give a red-violet diazo dye, detected by spectrophotometry at 550 nm.

### Plasmatic 8-Hydroxy-2-Deoxyguanosine (8-OHdG) Quantification

The plasmatic levels of 8-OHdG were measured using a competitive quantitative ELISA Kit (8-hydroxy-2-deoxyguanosine ELISA Kit, Abcam), according to manufacturer instructions, in a Synergy^TM^ multi-mode microplate reader (Gonçalves et al., 2017).

### *NFE2L2* and *KEAP1* Genes Expression Analysis

Total RNA was isolated from peripheral blood samples obtained from MDS patients using the Quick-RNA^TM^ MiniPrep (Zymo Research), according to the manufacturer’s instructions. Real-time quantitative PCR (qPCR) studies were performed to quantify the *NFE2L2* and *KEAP1* genes (normalized to *HPRT* gene). After extraction, total RNA was reverse transcribed into cDNA with SuperScript^TM^ III Reverse Transcriptase kit (Invitrogen, Life Technologies) using a 1:1 mix of random hexamers and oligo-dTs. Then, *NFE2L2, KEAP1*, and *HPRT* genes were amplified in duplicate using SsoFast^TM^ EvaGreen^®^ Supermixe (BioRad) in an IQ5 Real-Time PCR System (BioRad). To assess the reaction efficiency, standard curves were created for all studied genes using a serially diluted control sample. For each experiment was included a no template control (NTC) as the negative control. The specificity of qPCR reactions was confirmed using the melting curve analysis. The relative expression of the target genes was analyzed using the 2^Δ*Ct*^ formula.

### Statistical Analysis

Statistical analysis was performed using SPSS version 26.0, and graphics were constructed through GraphPad Prism version 6.0. Continuous variables were expressed as mean ± SEM (standard error of the mean), unless otherwise specified, and categorical variables as numbers and percentages. To account for changes in plasma lipid content, vitamin A and E were expressed in relation to cholesterol because lipids affect the concentration of these vitamins. All statistical analyses were two-sided, and a *p* < 0.05 was considered statistically significant. Normality was assessed by the Kolmogorov-Smirnov test. For normally distributed continuous variables, the Student’s *t*-test was performed to assess the statistical significance of the difference between means of ESA responders and non-responders. When continuous variables did not show normal distribution, the Mann-Whitney U was used. Logistic regression, adjusted to age and gender, was performed to establish the factors that were associated with ESA response. Factors that showed a significant association in the univariate analysis were included in the multivariate logistic regression to determine the associated independent variables. The calibration of logistic models was assessed by the Hosmer-Lemeshow goodness-of-the-fit test. Results from logistic analysis were expressed as adjusted odds ratios (OR) with the corresponding 95% confidence interval (CI). In this analysis, the OR corresponds to a 1-unit increase in the explanatory variable. Finally, receiver operating characteristic (ROC) curves were performed to evaluate the accuracy of significant parameters as ESA therapy response biomarker. The area under the curve (AUC) was calculated as a measurement of the accuracy of the test, and an optimal cut-off point was determined as the value of the parameter that maximized the sum of specificity and sensitivity (Youden’s J Index).

## Results

### Biodemographic and Clinical Characteristics of MDS Patients

The present study enrolled 66 patients diagnosed with MDS [median age of 74 years (range 22–89), 60.1% (*n* = 40) females and 39.9% (*n* = 26) males]. Table 1 show the biodemographical and clinical characteristics of MDS participants. MDS patients were diagnosed according to WHO classification (2016). Nine (13.6%) were diagnosed with MDS-SLD, 10 (15.2%) with MDS-RS, 40 (60.6%) with MDS-MD, and seven (10.6%) with MDS-EB. The IPSS-R prognostic score was lower in 38 (57.6%) patients, higher in 13 (19.7%), and not reported in 15 (22.7%). In fifteen MDS patients cytogenetic abnormalities were detected, having 37 patients good cytogenetic, 11 intermediate, and two poor. MDS patients had been transfused with a median of 24 U/l of sEPO, ranging from 4 to 494 U/l. From these patients, 44 (66.7%) received ESA-treatment as supportive care, and 20 (45.5%) did not respond to treatment. All patients that received ESA-treatment had sEPO levels below 500 U/l (median of 35 U/l, ranging from 4 to 494 U/l).

**TABLE 1:** Biodemographic and clinical characteristics of MDS patients.

**Characteristics**	**MDS (*n* = 66)**		**ESA-treated MDS (*n* = 44)**	
**Demographic features**				
**Gender (%)**				
Male	26	(39.9)	18	(40.9)
Female	40	(60.1)	26	(59.1)
Age (years)				
Median	74		79	
Range	22–89		47–87	
**Clinical features**				
**Hematological parameters (median, range)**				
WBC (×10^9^/l)	3.5	(1.3–13.0)	4.2	(1.3–13.0)
Hb (g/l)	10.6	(5.4–16.0)	9.9	(5.4–12.3)
Platelets (×10^9^/l)	98	(12–324)	107	(12–317)
Serum erythropoietin (U/l; median, range)	24	(4–494)	35	(4–494)
Serum ferritin (ng/ml; median, range)	237	(17–1750)	191	(28–1750)
Vitamin B12 (pg/ml; median, range)	608	(236–2000)	799	(236–2000)
Folic acid (ng/ml; median, range)	9.7	(2.2–24.0)	10.3	(4.0–24.0)
** WHO 2016 classification**				
MDS-SLD (%)	9	(13.6)	4	(9.1)
MDS-RS (%)	10	(15.2)	10	(22.7)
MDS-MD (%)	40	(60.6)	30	(68.2)
MDS-EB (%)	7	(10.6)	0	(0)
** IPSS-R risk groups**				
Lower-risk (IPSS-R score ≤ 3.5)	38		32	
Higher-risk (IPSS-R score > 3.5)	13		0	
Not recorded	15		12	
** Cytogenetics**				
Good	37		22	
Intermediate	11		10	
Poor	2		0	
Not recorded	15		12	

*MDS, myelodysplastic syndrome; ESA, erythropoietin-stimulating agents; WHO, World Health Organization; WBC, white blood cells; Hb, hemoglobin; MDS-SLD, MDS with single lineage dysplasia; MDS-MD, MDS with multilineage dysplasia; MDS-RS, MDS with ring sideroblasts; MDS-EB, MDS with excess blasts; IPSS-R, international prognostic scoring system revised.*

### Oxidative Stress Levels in ESA-Treated Patients

**FIGURE 1:**
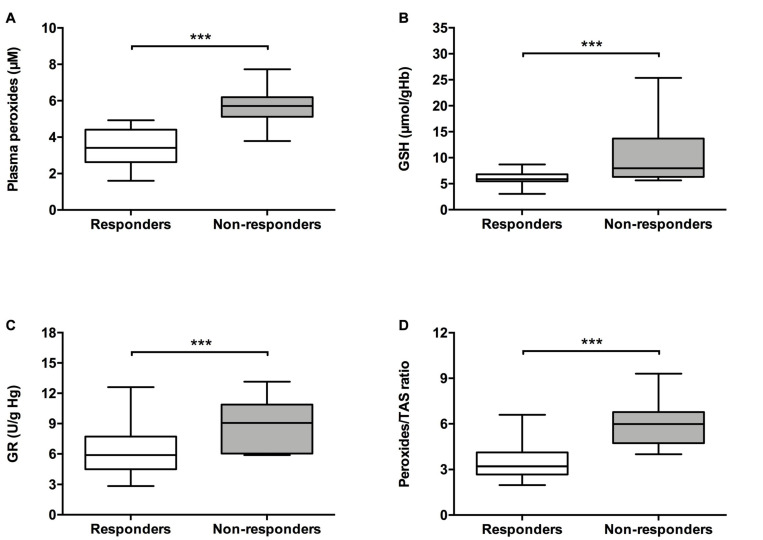
Analysis of oxidative stress parameters in patients with myelodysplastic syndrome according to their response to ESA treatment. The following oxidative stress levels are represented: **(A)** plasmatic peroxides, **(B)** erythrocyte reduced glutathione (GSH), **(C)** erythrocyte glutathione reductase (GR), and **(D)** peroxides/total antioxidant status (TAS) ratio. ***p ≤ 0.001.

To investigate the involvement of oxidative stress in the response to ESA-treatment, the plasmatic levels of reactive oxygen/nitrogen species (peroxides and NO), non-enzymatic antioxidant defenses [uric acid, vitamin E (plasmatic and erythrocytic), vitamin A, GSH, GSSG, TAS], enzymatic defenses (erythrocyte GPX and GR activities), and the levels of macromolecules oxidative damage [8-OH-dG and MDA (plasmatic and erythrocytic)] were compared between patients that responded and did not respond to ESA treatment (Figure 1 and Table 2). The peroxides/TAS and NO/TAS ratios were calculated to analyze the oxidative stress status of MDS patients. Moreover, we analyzed the expression of *NFE2L2* gene, that encode the transcription factor NRF2 (a redox regulator), and its negative regulator, the *KEAP1* gene.

**TABLE 2:** Analysis of oxidative stress parameters in patients with myelodysplastic syndrome according to ESA response treatment.

**Parameter**	**ESA responders**		**ESA non-responders**		***p*-value**
	**Mean**	**SEM**	**Mean**	**SEM**	
Uric acid (mg/dl)	0.40	0.03	0.40	0.04	0.944
pVitamin A (μM/mM)	0.45	0.04	0.43	0.03	0.962
pVitamin E (μM/mM)	6.65	0.31	6.02	0.30	0.203
eVitamin E (nmol/gHb)*	23.5	16.8	46.6	60.4	0.059
pMDA (μM)	0.84	0.05	0.75	0.05	0.190
eMDA (μmol/g Hb)	81.6	8.5	53.3	4.8	0.268
NO (μM)	11.8	1.2	13.1	1.5	0.450
eGPx (U/g Hb)*	4.50	4.42	4.85	6.46	0.409
8-OH-dG (ng/ml)	34.2	1.4	39.7	1.0	0.069
TAS (mM)*	1.04	0.29	0.96	0.12	0.257
NO/TAS ratio*	14.4	14.0	13.3	7.9	0.723

*These values are represented as median and interquartile range. Plasmatic vitamin A and E values are represented as vitamin A:cholesterol and vitamin E:cholesterol ratios, respectively. p, plasmatic; e, erythrocytic; 8-OH-dG, 8-hydroxy-2′-deoxyguanosine; NO, nitric oxide; GSH, reduced glutathione; TAS, total antioxidant status; MDA, malondialdehyde; SEM, standard error of the mean.

**FIGURE 2:**
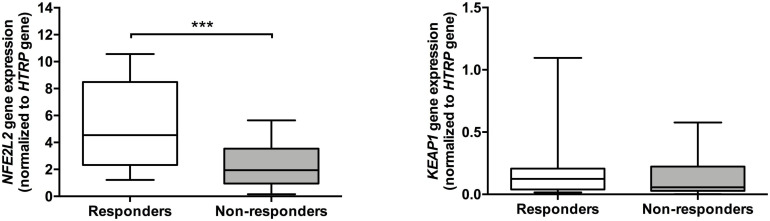
Expression of *NFE2L2* and *KEAP1* genes in patients with myelodysplastic syndrome according to their response to ESA treatment. The RNA expression for *NFE2L2* and *KEAP1* genes were normalized to the *HPRT* gene (endogenous control). ***p ≤ 0.001.

As shown in Figure 1, patients that respond to ESA treatment showed lower levels of plasma peroxides (3.48 ± 0.21 μM; *p* < 0.001), cellular GSH [median (Med): 5.93, interquartile range (IqR): 1.39 μmol/g Hb; *p* < 0.001], and cellular GR activity (Med: 5.89, IqR: 3.39 U/g Hb; *p* = 0.001) in comparison to patients who did not respond to ESA treatment (peroxides: 5.65 ± 0.21 μM; GSH: Med: 7.99, IqR: 7.39 U/g Hb; GR: Med: 9.06, IqR: 4.83 U/g Hb). ESA responders also showed lower levels of peroxides/TAS ratio (3.60 ± 0.24, *p* < 0.001) compared to those without response (6.17 ± 0.38 μM). Moreover, MDS patients that respond to ESA treatment showed higher expression levels of the *NFE2L2* gene (Med: 4.544, IqR: 6.150; *p* = 0.001) than those that did not respond to ESA treatment (Med: 1.945, IqR: 2.590; Figure 2). No differences were observed between MDS subtypes.

### Oxidative Stress Levels as Predictive Biomarker of ESA Treatment Response

The association between oxidative stress parameters and ESA response was analyzed through logistic regression (Table 3). The levels of plasmatic peroxides, the peroxides/TAS ratio, the cellular GSH and GR activity were significantly associated with ESA treatment in the univariate analysis. In the multivariate analysis, peroxides were significantly associated with ESA non-response [Odds ratio (OR): 0.111; 95% confidence interval (CI): sensitivity: 92%; specificity: 90%; positive predictive value (PPV): 96%; negative predictive value (NPV): 80%; *p* = 0.007]. We did not find any association between sEPO levels and the response to ESA treatment.

**TABLE 3:** Significant oxidative stress parameters as ESA response biomarker in myelodysplastic syndrome.

**Biomarkers**	**AUC**		**Cut-off**				
	**value (95% CI)**	***p-*value**	**value**	**SEN (%)**	**SPE (%)**	**PPV (%)**	**NPV (%)**
Peroxide (μM)	0.946 (0.883–1.000)	< 0.001	>4.96	80	100	100	86
GR (U/g Hb)	0.793 (0.661–0.924)	0.001	>5.97	90	58	64	88
GSH (μmol/g Hb)	0.814 (0.690 –0.937)	< 0.001	>8.86	45	100	100	69
Peroxides/TAS ratio	0.918 (0.838–1.000)	< 0.001	>3.94	100	75	77	100
*NFE2L2* gene expression	0.785 (0.653–0.918)	0.001	<4.23	90	54	62	87

*OR, odds ratio; CI, confidence interval; GR, glutathione redutase; GSH, reduced glutathione; TAS, total antioxidant status; AUC, area under the curve; CI, confidence interval; SEN, sensitivity; SPE, specificity; PPV, positive predictive value; NPV, negative predictive value.*

**FIGURE 3:**
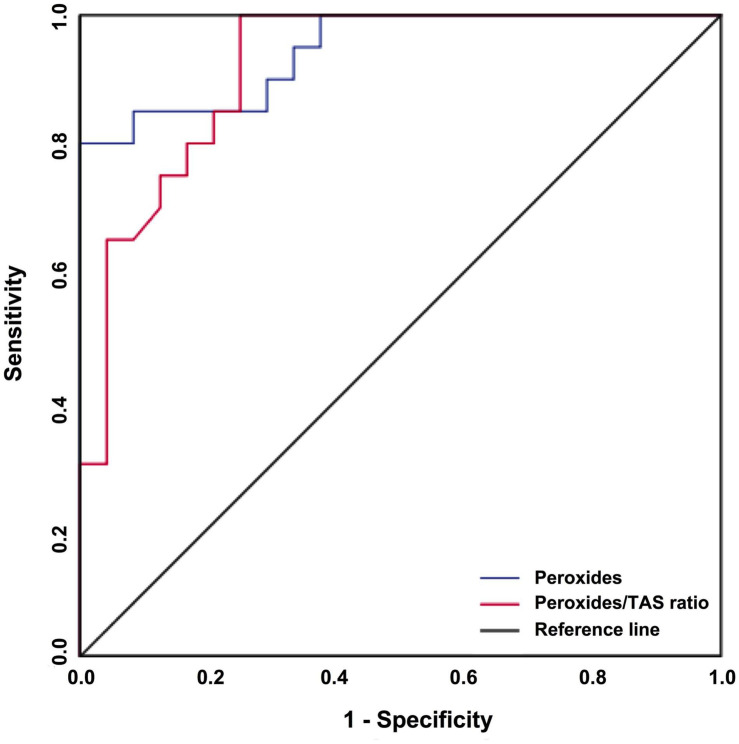
The predictive value of peroxides and peroxides/TAS ratio regarding ESA treatment responses in MDS patients. A ROC curve analysis for peroxides and peroxides/TAS ratio was performed to assess its potential as a biomarker of ESA treatment response.

The potential of oxidative stress parameters as predictive biomarkers of response to ESA treatment was assessed by ROC curves (Table 4 and Figure 3). The plasmatic peroxides levels and the peroxides/TAS ratio were the most accurate biomarker for ESA response, with an area under the curve (AUC) of 0.946 [95% CI: 0.883–1.000; *p* < 0.001] and 0.918 (95% CI: 0.838–1.000; *p* < 0.001), respectively. The peroxides levels higher than 4.96 μM (sensitivity: 80%; specificity: 100%; PPV: 100%; NPV: 86%) and the peroxides/TAS ratio higher than 3.94 (sensitivity: 100%; specificity: 75%; PPV: 77%; NPV: 100%) were defined as the optimal cut-off values for identify ESA non-responder MDS patients. The GR activity, the GSH levels and the *NFE2L2* gene expression also showed potential as ESA response biomarkers. Despite its lower potential, the GSH erythrocytes levels (AUC = 0.814; 95% IC: 0.690–0.937; *p* < 0.001) show to be also good biomarkers of ESA response. The best GSH cut-off values were 8.86 μmol/g Hb (sensitivity: 45%; specificity: 100%; PPV: 100%; NPV: 69%). The GR activity (AUC: 0.793; 95% CI: 0.661–0.924; *p* = 0.001; sensitivity: 90%; specificity: 58%; PPV: 64%; NPV: 88%) and the *NFE2L2* gene expression levels (AUC: 0.785; 95% CI: 0.653–0.918; *p* = 0.001; sensitivity: 90%; specificity: 55%; PPV: 62%; NPV: 87%) were considered the biomarkers with lower potential to predict ESA response.

**TABLE 4:** Predictive potential of oxidative stress parameters as biomarkers of ESA response.

**Biomarkers**	**Univariate**		**Multivariate**	
	**OR (95% CI)**	***p-*value**	**OR (95% CI)**	***p-*value**
Peroxide (μM)	0.088 (0.021–0.371)	0.001	0.137 (0.024–0.787)	0.026
GR (U/g Hb)	0.519 (0.315–0.855)	0.010	0.207 (0.018–2.447)	0.211
GSH (μmol/g Hb)	0.644 (0.477–0.870)	0.004	0.631 (0.310–1.283)	0.203
Peroxide/TAS ratio^§^	0.252 (0.117–0.541)	0.001	–	–
*NFE2L2* gene expression	1.707 (1.177–2.477)	0.005	3.555 (0.848–14.900)	0.830

*^§^Due to multicollinearity between peroxide and peroxide/TAS ratio, the last one was not included in multivariate analysis. OR, odds ratio; CI, confidence interval; GR, glutathione redutase; GSH, reduced glutathione; TAS, total antioxidant status.*

## Discussion

The current study aimed to understand better the mechanisms of resistance to ESA therapy and the influence of oxidative stress in treatment response in lower-risk MDS patients. We found that patients who respond to ESA treatment have lower levels of plasma peroxides, cellular GSH, cellular GR activity, peroxides/TAS ratio, and higher levels of *NFE2L2* gene expression in comparison to patients who did not respond to ESA treatment. These parameters are good biomarkers of therapy response, with peroxides levels being the best and accurate biomarker of ESA response. To the best of our knowledge, only one study analyzed the association between oxidative stress and ESA response. In this study, Khalil et al. (2016) found that patients with an end-stage renal disease with lower erythrocyte superoxide dismutase and higher MDA levels show poor response to ESA treatment (Khalil et al., 2016). Here we found that response to ESA treatment is associated with a decrease in peroxides levels and peroxides/TAS ratio and a decrease in GSH levels and GR activity. Although we observed an increase in these antioxidant defenses on non-responders. Globally these patients presented a oxidative stress state that is in accordance with other studies that show that oxidative stress is associated with drug resistance in cancer (Pour Khavari et al., 2018; Maurya et al., 2021). ROS is known for its ability to induce mutations and promote cancer cell growth and anticancer drug resistance. The oxidative stress in cancer cells has been shown to correlate with the aggressiveness of tumors and poor survival of patients with cancer (Trachootham et al., 2009). Oxidative stress is exceptionally important to erythropoiesis and is involved in proliferation, survival, and differentiation of erythroid cell progenitors in response to EPO binding to erythropoietin receptor, and can also induce cell death by apoptosis (Ghaffari, 2008). Since oxidative stress has deleterious effects on erythroid progenitor cells, the increase in oxidative stress ratio and peroxides could induce erythrocytes apoptosis/inefficient erythropoiesis and, therefore, justify the non-responder phenotype observed in this study. Additionally, the transcription factor NRF2 is considered a prognostic biomarker in cancer, with very high levels associated with poor response to anticancer drugs (Frijhoff et al., 2015). However, we found that MDS patients that did not respond to ESA treatment have lower levels of *NFE2L2*, the gene that encode the NRF2 transcription factor. This result is in agreement with the higher levels of peroxides and oxidative stress ratio observed in these patients. Furthermore, we previously found that oxidative stress parameters and the Δψ_*mit*_ are diagnostic biomarkers and survival predictors for MDS, with GSH levels providing the most accurate and reliable indicator of MDS diagnosis and survival (Gonçalves et al., 2015). Additionally, in another study we found that GSH levels correlate with the relapse and survival of acute lymphoblastic leukemia patients (Sarmento-Ribeiro et al., 2012).

Several studies have examined factors, in lower-risk MDS patients, that could be used to predict response to ESA treatment and to shape treatments more efficiently. One of the most studied factors in response to ESAs associated with or without G-CSF is the sEPO levels, and numerous studies have reported correlations between sEPO and therapy response. The majority of these studies used a sEPO cutoff of 100 U/L, with response rates ranging from 50 to 93% for patients with sEPO < 100 U/L *versus* 12–58% for patients with sEPO > 100 U/L (Park et al., 2020). In the present study we did not find any association between sEPO and ESA response, but the response rates were 58% for patients with sEPO < 100 U/L and 38% for patients with sEPO > 100 U/L. However, in a previous study including 102 MDS patients, we found that sEPO is a predictive factor for response to therapy with subcutaneous EPO (Cortesão et al., 2015). Several other factors have also been studied including absolute neutrophil count (Stasi et al., 2005), cytopenia levels (Molteni et al., 2013), hemoglobin levels (Stasi et al., 2005; Santini et al., 2013; Houston et al., 2017), platelet count (Stasi et al., 2005; Santini et al., 2013), age (Stasi et al., 2005; Houston et al., 2017), gender (Stasi et al., 2005; Houston et al., 2017), burst-forming unit-erythroid levels (Frisan et al., 2010), bone marrow blasts (Stasi et al., 2005; Frisan et al., 2010; Santini et al., 2013), IPSS status (Stasi et al., 2005; Frisan et al., 2010; Santini et al., 2013), *p*-ERK1/2 levels (Frisan et al., 2010), serum TNF-alpha (Stasi et al., 2005), somatic mutations (Kosmider et al., 2016), among others.

This work has some limitations that must be taken into account. We recruited almost all patients newly diagnosed with MDS during recruitment time, but we were only able to study a relatively small cohort of patients, especially those treated with ESA. The oxidative stress parameters were only analyzed in peripheral blood samples (plasma, total leucocytes, and/or erythrocytes). Although MDS is a clonal stem cell disorder, the same studies must be repeated in erythroid precursor cells obtained from bone marrow samples. However, this fact may also be one of the work strengths, since peripheral blood is a more accessible and less invasive biological sample.

In conclusion, the present report demonstrated a correlation between oxidative stress levels and the response to ESA treatment in lower-risk MDS patients. We found that peroxides levels and peroxides/TAS ratio are good and accurate peripheral biomarkers that predict patients that will not respond to ESA therapy.

## Data Availability Statement

The raw data supporting the conclusions of this article will be made available on request to the corresponding author.

## Ethics Statement

The studies involving human participants were reviewed and approved by Ethics Committee of the Faculty of Medicine of the University of Coimbra Azinhaga de Santa Comba, Coimbra, Portugal. The patients/participants provided their written informed consent to participate in this study.

## Author Contributions

AG and AS-R conceived the study. AG, RA, IB, AP, and JJ performed the experiments. EC and BM recruited and collected patient data. AG and BO analyzed and interpreted the data. AG drafted the manuscript. AS-R and JN reviewed and edited the manuscript. All authors read and approved the final manuscript.

## Conflict of Interest

The authors declare that the research was conducted in the absence of any commercial or financial relationships that could be construed as a potential conflict of interest.
